# Trends in Suicide Rates by Race and Ethnicity in the United States

**DOI:** 10.1001/jamanetworkopen.2021.11563

**Published:** 2021-05-26

**Authors:** Rajeev Ramchand, Joshua A. Gordon, Jane L. Pearson

**Affiliations:** 1 National Institute of Mental Health, Rockville, Maryland

## Abstract

This cross-sectional study examines trends in the suicide rate among racial and ethnic subgroups in the United States from 1999 to 2019.

## Introduction

The US Centers for Disease Control and Prevention reported a decrease in the 2019 suicide rate, from 14.2 per 100 000 individuals in 2018 to 13.9 per 100 000 individuals in 2019, representing 833 fewer suicides.^1^ This is the first national year-over-year decrease since 1999, but efforts need to consider trends among subgroups. Growing concerns regarding increasing rates of suicides among Black youth^2^ led us to examine subgroup trends over the past 2 decades.

## Methods

This cross-sectional study did not require institutional review board approval or informed consent because it used public mortality data, per the Common Rule. We adhered to the Strengthening the Reporting of Observational Studies in Epidemiology (STROBE) reporting guideline.

Data for this study came from the National Vital Statistics System (NVSS).^3^ NVSS age-adjusted rates are based on postcensal population estimates. Mortality, race, and ethnicity data derive from death certificates and include 4 race categories (White, Black, American Indian or Alaska Native, and Asian or Pacific Islander) and 3 ethnicity categories (Hispanic or Latino, not Hispanic or Latino, and not stated). Population race/ethnicity data are the bridge race categories created by the National Center for Health Statistics and the Census Bureau. Time-trend regression models, performed with Stata version 16.1 (StataCorp) with time coefficient 95% CIs were used to determine whether the 2019 rate was expected (ie, within the interval created by adding the lower and upper confidence limits to the 2018 rate).

## Results

**Figure 1.**
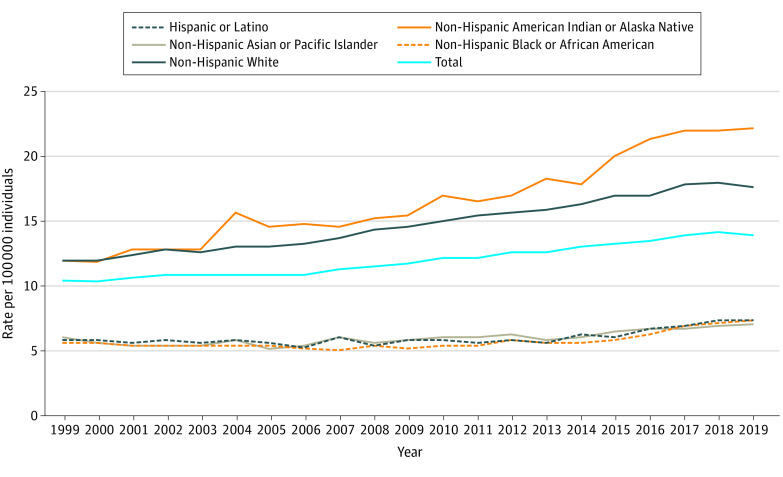
Age-Adjusted Suicide Rates by Race/Ethnicity, 1999 to 2019

The overall age-adjusted rates (Figure 1) indicate a decrease in suicide rates between 2018 and 2019 for White and American Indian or Alaska Native individuals, reflecting the decrease in total deaths. The age-adjusted rate increased for Black and Asian or Pacific Islander individuals. The increasing trend for these groups began in 2014; between 2014 and 2019, the suicide rate increased by 30% for Black individuals (from 5.7 to 7.4 per 100 000 individuals) and 16% for Asian or Pacific Islander individuals (from 6.1 to 7.1 per 100 000 individuals). Based on the 2014 to 2017 trend, the change between 2018 to 2019 was in the expected range for Black individuals (2019 actual: 7.4 per 100 000 individuals; expected: 7.3-8.0 per 100 000 individuals), Asian or Pacific Islander individuals (2019 actual: 7.1 per 100 000 individuals; expected: 7.0-7.5 per 100 000 individuals), and Hispanic individuals (2019 actual: 7.3 per 100 000 individuals; expected: 7.3-8.0 per 100 000 individuals), but the change was outside of the expected range for White individuals (2019 actual: 17.6 per 100 000 individuals; expected: 18.0-18.9 per 100 000 individuals), American Indian or Alaskan Native individuals (2019 actual: 22.2 per 100 000 individuals; expected: 22.5-24.6 per 100 000 individuals), and the total population (2019 actual: 13.9 per 100 000 individuals, expected: 14.3-14.7 per 100 000 individuals).

**Figure 2.**
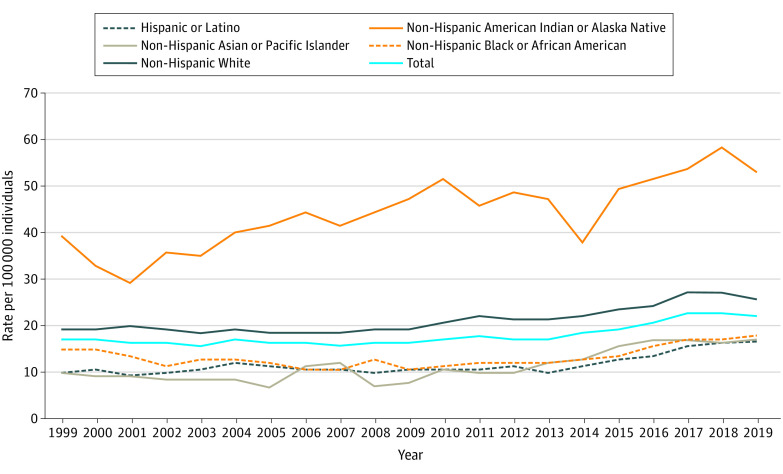
Crude Suicide Rate Among Male Youth Aged 15 to 24 Years by Race/Ethnicity, 1999 to 2019

For both male and female youth aged 15 to 24 years, suicide rates were higher among American Indian or Alaskan Native youth (eg, 2019 rate among female youth: 23.0 per 100 000 individuals) and White youth (6.1 per 100 000 individuals) relative to Black youth (4.3 per 100 000 individuals), Asian or Pacific Islander youth (5.1 per 100 000 individuals), and Hispanic youth (4.4 per 100 000 individuals) for all years. Figure 2 illustrates that the suicide rate increased for Black male youth in this age group by 47%, from 12.2 per 100 000 individuals in 2013 to 17.9 per 100 000 individuals in 2019, and for Asian or Pacific Islander male youth in this age group by 40%, from 12.0 per 100 000 individuals in 2013 to 16.8 per 100 000 individuals in 2019. The rates from 2018 to 2019 increased for Black and Asian or Pacific Islander male youth in this age group within the expected range given the 2013 to 2017 trend (Black, 2019 actual: 17.9 per 100 000 individuals; expected: 17.5-19.0 per 100 000 individuals; Asian or Pacific Islander, 2019 actual: 16.8 per 100 000 individuals; expected: 16.5-17.9 per 100 000 individuals), in contrast to the decrease seen among White youth in this age group, which fell outside of the expected range (2019 actual: 25.4 per 100 000 individuals; expected: 27.7-29.0 per 100 000 individuals). Similar increases were found among female youth aged 15 to 24 years: between 2013 and 2019, the rate increased by 59% among Black female youth in this age group (from 2.7 to 4.3 per 100 000 individuals) and 42% among Asian or Pacific Islander female youth in this age group (from 3.6 to 5.1 per 100 000 individuals).

## Discussion

The data analyzed here came from the official source of US mortality data; nonetheless, misclassification of death by suicide and race/ethnicity are limitations. Examining suicide trends in subgroups is necessary to inform prevention efforts that reach everyone. The COVID-19 pandemic has increased mental health awareness. Although we will not be able to examine the association of COVID-19 with suicide rates for some time, recent reports suggest racial disparities in COVID-19 outcomes^4^ and suicide deaths in 1 state,^5^ and the increase in Black and Asian or Pacific Islander youth suicide rates are worrisome. Efforts are needed to mitigate suicide and its risk factors in population subgroups, which may include systemic and other factors that have placed increased stress on individuals who belong to racial/ethnic minority groups, particularly Black and Asian or Pacific Islander individuals. The National Action Alliance for Suicide Prevention’s Mental Health and Suicide Prevention National Response to COVID-19 has identified actions needed to reduce suicide and mental disorders now and after the pandemic ends, including investments in equitable delivery of mental health and suicide preventive services and approaches that treat the root causes of suicide and mental illness.^6^ Research conducted in concert with stakeholders from population subgroups is needed to identify and mitigate causes of suicidal distress and enhance equitable access and effectiveness of prevention efforts.
